# Engineered eMutS based magnetic separation system for high-fidelity DNA synthesis

**DOI:** 10.1016/j.synbio.2026.02.009

**Published:** 2026-03-06

**Authors:** Xiaohang Wang, Yefei Wang, Wei Wang, Wenshuang Liu, Hui Jia, Mengsha Li, Mingying Li, Changle Wu, Jian Xu, Xiang Sheng, Jia Zhang

**Affiliations:** a Key Laboratory of Photoelectric Conversion and Utilization of Solar Energy, Qingdao New Energy Shandong Laboratory, Qingdao Institute of Bioenergy and Bioprocess Technology, Chinese Academy of Sciences, Qingdao, Shandong, China; b State Key Laboratory of Engineering Biology for Low-Carbon Manufacturing, Tianjin Institute of Industrial Biotechnology, Chinese Academy of Sciences, Tianjin, China; c University of Chinese Academy of Sciences, Beijing, China; d Shandong Energy Institute, Qingdao, Shandong, China; e National Center of Technology Innovation for Synthetic Biology, Tianjin, China; f University of Jinan, Jinan, Shandong, China

**Keywords:** DNA synthesis error correction, Engineered MutS, Molecular dynamics simulations, Biotin-streptavidin system, Magnetic bead separation, High-throughput workflow

## Abstract

DNA synthesis fidelity remains a critical bottleneck in large-scale gene assembly. Although conventional post-synthetic purification methods (e.g., HPLC, PAGE) and enzymatic mismatch-cleavage strategies offer partial solutions, they often suffer from limited throughput, high cost, and incompatibility with automated workflows. To overcome these limitations, we developed an integrated, sensitive and high-throughput error-correction platform that combines a rationally engineered *Escherichia coli* MutS protein (eMutS) with a biotin-streptavidin magnetic-bead separation system (eMBS). Specifically, a single point mutation (E38N) was introduced into eMutS to enhance mismatch-recognition specificity while suppressing non-specific binding to perfectly matched DNA by optimizing key residue-base interactions of enhanced π-π stacking and direct hydrogen bond formation. Two cysteine mutations (L157C/G233C) were further incorporated to promote disulfide bond formation, which strengthens the affinity toward all three error types and enhances structural stability. The resulting eMutS-L157C/G233C-E38N-biotin fusion protein which was immobilized onto streptavidin-coated magnetic beads, enables rapid and efficient magnetic separation of error-containing heteroduplexes from error-free homoduplexes. In terms of superior performance with mixed DNA substrates, eMBS reduces total errors by 13.1∼63.3% (n = 3 independent experiments) in pools starting with 73.6∼98.6% correct sequences, and achieves an error-free DNA ratio of 99.1%. By eliminating labor-intensive electrophoresis or column purification steps, this sensitive, versatile and high-throughput workflow can support high-fidelity DNA synthesis.

## 1 Introduction

Synthetic biology, characterized by the engineered-driven design and construction of synthetic genomes, is widely regarded as the third revolution in biotechnology and landmark discoveries of the DNA double helix and the completion of the Human Genome Project [1]. Within this field, gene synthesis serves as a foundational enabling technology [2], with the global market projected to reach $5.78 billion by 2030 [3]. As the comprehension of biological systems has advanced, the redesign and de novo creation of genomic constructs have become among the most imaginative and dynamic areas of biological research [4]. Through precise DNA design and synthesis, it enables researchers to reprogram cellular functions and even create artificial life, significantly advancing our capacity to understand, predict and manipulate living organisms [5].

Despite their transformative potential, current gene synthesis workflows suffer from pervasive errors introduced during oligonucleotide synthesis, assembly, and amplification [6]. The predominant method for synthesizing short oligonucleotides relies on solid-phase phosphoramidite chemistry, which is conducted on controlled pore glass via a four-step cycle process [7,8]. However, this approach involves imperfect chemical reactions that introduce errors, especially in longer sequences, and is characterized by high cost and low throughput [7]. In contrast, DNA microarrays or microfluidic chips provide a more cost-effective and high-throughput alternative, enabling large-scale parallel synthesis of oligonucleotide pools [9]. Nonetheless, these technologies are constrained by higher error rates than conventional column-based methods are. The absence of inherent mismatch repair mechanisms during in vitro gene synthesis results in low fidelity, with error rates typically ranging from 10^−2^ to 10^−3^ (or 1 to 10 errors per kb) [6].

The origins of errors in gene synthesis are multifaceted. *In column-based synthesis*, acidic reagents can cause depurination, leading to erroneous incorporations [10], whereas incomplete deprotection or inefficient capping contributes to insertion and deletion errors [11]. *In microarray-based synthesis*, improper reagent dispensing or storage conditions further introduce errors [11]. Additionally, errors accumulate during fragment assembly (repeated thermal cycling during PCR inflicts structural damage on DNA, and the intrinsic error rate of DNA polymerase introduces mutations) [12,13]. As the length of synthetic DNA constructs increases, errors such as base substitutions, insertions, and deletions accumulate progressively. This reduces the yield of accurate full-length products, amplifies the workload for screening, and elevates overall costs [14,15]. Collectively, these challenges diminish the practical yield of error-free constructs, escalate downstream verification expenses, and hinder the reliable construction of large synthetic DNA molecules. Therefore, integrating efficient error-correction strategies into gene synthesis workflows has become crucial for enhancing the fidelity and scalability of synthetic DNA production.

To address these issues, a range of post-synthetic purification strategies including high-performance liquid chromatography (HPLC) [16], polyacrylamide gel electrophoresis (PAGE) [10], desalting, and column purification based on hydrophobic protecting groups [6], have been widely adopted to enrich full-length, error-free oligonucleotides. While these methods improve quality by removing impurities and mitigating adverse effects in subsequent assembly, they cannot ensure absolute sequence accuracy and often involve considerable operational complexity. Consequently, enzymatic error-correction methods have garnered increasing attention. These approaches employ enzymes capable of recognizing, binding to, or excising structural distortions in the DNA double helix caused by mispaired bases [[17], [18], [19]]. Existing enzymatic systems fall into two main categories: (*i*) *Enzymatic mismatch cleavage (EMC) platforms*, which integrate exonucleases and endonucleases such as T7 endonuclease I [20], Surveyor nuclease [21] and the CorrectASE kit [22]. These systems typically involve endonuclease recognition and cleavage at mismatch sites, followed by exonuclease-mediated excision [[23], [24], [25]]. However, challenges remain due to the high cost and limited efficiency of individual enzymes. (*ii*) *MutS-based mismatch recognition*, which exploits the innate ability of the MutS protein to specifically bind double strand DNA mismatches and form stable DNA-protein complexes. These complexes can be separated from error-free DNA via electrophoresis or solid-phase adsorption [13,26]. MutS is a key component of the conserved DNA mismatch repair (MMR) system, which also includes MutL and MutH [27]. Different MutS homologs exhibit distinct DNA-binding properties [25,28]; for instance, *Thermus aquaticus* MutS (tMutS) is thermostable [29], but has a lower mismatch affinity than *Escherichia coli* MutS (eMutS), resulting in reduced correction efficiency [[30], [31]]. Thus, eMutS is often preferred in synthetic applications. Among the methods for isolating MutS error-containing DNA complexes from error-free DNA, electrophoresis is cumbersome [26], whereas immobilized MutS systems offer advantages in throughput, automation compatibility, and cost efficiency [32].

In previous work, we engineered eMutS through rational protein and process design, extending its functional shelf life from 7 to 63 days [9]. Our Improved MutS Immobilized Cellulose Column (iMICC) method achieved a 6.6-fold improvement in base accuracy and a 26.7-fold cost reduction compared with the CorrectASE system [9]. Despite these advances, two major limitations hinder its broad adoption: nonspecific DNA binding and labor-intensive manual procedures. Specifically, the iMICC method requires column recovery, which consumes substantial sample volume involves labor-intensive steps and prolongs processing time, complicating automation integration [26]. To overcome these drawbacks, we pursued two strategic improvements: rational protein redesign to minimize nonspecific binding, and a shift to magnetic bead-based separation to streamline operation and facilitate automation. To address nonspecific binding (a major challenge in specificity), we employed a rational design strategy. This approach leverages bioinformatics tools and comparative sequence analysis across homologs to identify key amino acid residues for targeted mutagenesis [33]. Through site-directed mutagenesis, these residues are modified to generate variants with enhanced binding specificity [[34], [35]]. Rational design is particularly powerful for tailoring enzyme specificity and has demonstrated significant improvements in various targeted applications [[36], [37]]. To replace the cumbersome column-based recovery, magnetic bead-based separation offers an attractive alternative. Benefiting from a high surface area, rapid magnet-assisted retrieval, and innate compatibility with robotic liquid-handling systems, magnetic beads are far more suitable than traditional fiber columns as solid-phase carriers for MutS in DNA mismatch repair applications. The biotin-streptavidin system, renowned for its exceptionally strong non-covalent interaction (Kd ≈ 4 × 10^−14^ M) [38], is widely used in molecular and cellular research due to its high specificity and affinity [38]. Recent studies have demonstrated a novel mutation detection method using biotinylated MutS coupled with streptavidin-coated magnetic beads, confirming that the MutS-biotin fusion retains effective mismatch binding activity in solution [39]. Furthermore, a biotin-streptavidin bridge oligonucleotide aptamer structure has been developed for high-specificity target protein recognition [40]. These findings confirm that biotinylation enables efficient capture and detection via streptavidin without significantly compromising MutS's native DNA mismatch-binding capability in vitro [39]. However, these prior applications focused primarily on mutation detection and protein recognition rather than error correction, leaving the potential of the MutS-biotin-streptavidin system for improving gene synthesis fidelity largely unexplored.

Here, we overcome the key limitations of current error-correction technologies by developing an integrated engineered eMutS coupled with a biotin-streptavidin magnetic-bead separation system (eMBS) that simultaneously enhances specificity and compatibility with automated workflows. Specifically, we: (*i*) rationally redesigned eMutS to enhance the mismatch recognition specificity, improving its binding affinity to mismatched DNA while reducing non-specific binding to correctly matched DNA; (*ii*) performed an atomistic dissection of the MutS mismatch identification mechanism through multiscale theoretical simulations, identifying key residues and structural motifs governing fidelity; and (*iii*) introduced a magnetic bead-based workflow that eliminates column-based recovery and minimizes manual intervention. The resulting platform achieves superior fidelity in DNA synthesis while meeting the high-throughput and automation requirements of modern synthetic biology applications.

## 2 Material and methods

### 2.1 Reagents and strains

All chemicals used were of analytical grade or higher. Luria-Bertani (LB) medium (10 g/L tryptone, 10 g/L NaCl, 5 g/L yeast extract), isopropyl β-d-1-thiogalactopyranoside (IPTG), and ampicillin were purchased from Sangon Biotech Co. (Shanghai, China). All restriction enzymes and T4 DNA ligase were obtained from Thermo Fisher Scientific Inc. (MA, United States). KOD Plus DNA polymerase was supplied by TOYOBO (Osaka, Japan), and Q5 High-Fidelity DNA polymerase was obtained from New England Biolabs (NEB, MA, Ipswich, United States). *Escherichia coli* strains BL21(DE3) and DH5α, along with the plasmid pET-21c (+) were obtained from Sangon Biotech (Shanghai, China). LB medium, supplemented with 15 g/L agar for solid plates, was used for aerobic cultivation of all strains. *E. coli* DH5α and BL21(DE3) were employed for cloning and recombinant protein expression, respectively. Plasmid pET-21c (+) served as the vector for expressing fusion proteins. All oligonucleotides were synthesized by Sangon Biotech (Shanghai, China).

### 2.2 Computational design and mismatch recognition analysis of point mutations

To design eMutS point mutations for enhanced mismatch-recognition specificity, molecular dynamics (MD) simulations and Molecular Mechanics/Poisson-Boltzmann Surface Area (MM/PBSA) free energy calculations were performed [41]. The DNA-enzyme binary complex was modeled by replacing the DNA in the crystal structure of the eMutS-DNA complex (PDB ID: 1E3M) [42] with the specific DNA used in the current experiments. Structures of the mutants were obtained using the Scwrl4 program [43]. The protonation state of all titratable residues (His, Asp and Glu) were assessed by using the propka server [44], and the position of the hydrogen atoms on histidine was inspected considering the surrounding H-bonding network. All histidine residues were in their neutral state and all acidic residues were set as deprotonated state. The AMBER force fields FF19SB and DNA.bsc1 were used for protein and DNA, respectively. And, Mg^2+^ ions were described using frcmod.ions234lm_1264_tip3p force field. The system was solvated in a TIP3P water [45,46] box that extended from the protein boundary of 12.0 Å, and then Na^+^ ions were added to neutralize the system.

After the system was setup, the resulting system was subjected to two-step minimization to remove the unreasonable interatomic contacts. At first, the protein, DNA and Mg^2+^ were restrained using a force constant of 20 kcal (mol^−1^ Å^−2^), while the solvent and Na^+^ ions in the system were fully optimized (2500 steps for the steepest descent and 2500 steps for the conjugate gradient). For the second step of minimization, the parameters used are the same as the first step, but the simulation was performed without any restraint. Subsequently, the system was slowly heated from 0 to 300 K under the NVT ensemble for 200 ps with a 2 fs time step, with a restraint constant of 50 kcal (mol^−1^ Å^−2^) on the protein, DNA and Mg^2+^ to avoid any unreasonable movement. Then the density of the system was equilibrated for 250 ps under the NPT ensemble at a constant temperature of 300 K and pressure of 1.0 bar. During this process, a Langevin thermostat [47] with a collision frequency of 2 ps^−1^ and a Berendsen barostat [48] with a pressure relaxation time of 1 ps were used.

As to computational design of point mutation, a 10 ns productive MD simulation under the NPT ensemble was performed with a time step of 2.0 fs, the periodic boundary conditions were employed throughout the simulations. A Particle Mesh Ewald (PME) [49] method with a cutoff distance of 10 Å for non-bonded interactions was used, and the bonds involving hydrogen were constrained by using a SHAKE [50] method. The trajectories after 4 ns were used for the MM/PBSA calculation of binding energies (Δ*G*). The internal, electrostatic and van der Waals contributions were obtained from force field. The Poisson-Boltzmann (PB) equation was employed to calculate the polar desolation energies, whereas the internal and external dielectric constants were set to 4.0 and 80.0 respectively. The solvent accessible surface area was adopted to calculate nonpolar solvation energies, with a solvent probe radius of 1.4 Å.

As to the best mutation confirmed experimentally, a 50 ns productive MD simulation under the NPT ensemble was performed to elucidate the structural and energetic basis of mismatch recognition. The calculated root-mean-squared deviations (RMSDs) of the trajectories from the MD simulations of wild-type (WT) enzyme and the eMutS-L157C/G233C-E38N-biotin mutant toward three types of error-containing DNA substrates (insertion, deletion, and substitution) are shown in Fig. S1. One can see that all systems are well converged. All the productive MD simulations were performed using the GPU version of the AMBER 22 software package [51].

### 2.3 Plasmids for expression of various fusion proteins

The expression plasmid for eMutS-L157C/G233C derived from *E.coli*, was constructed in our previous study [9]. In the present study, eight plasmids were constructed on the basis of the eMutS-L157C/G233C backbone, as summarized in Table S1. Each construct encodes a fusion protein featuring an N-terminal biotin tag and enhanced green fluorescent protein (EGFP), along with a C-terminal 6 × His tag. The biotin moiety enables high-affinity binding to streptavidin, while EGFP serves as a visual reporter for monitoring expression and purification. The 6 × His tag facilitates purification via immobilized metal affinity chromatography (IMAC). Streptavidin-coated magnetic beads were obtained from Sangon Biotech Co. (Shanghai, China).

### 2.4 Recombinant expression and purification of variants in *E.coli*

Constructed plasmids were transformed into *E. coli* BL21(DE3) cells for recombinant protein expression using established protocols [9]. Transformants were cultured overnight, and 2 mL of this culture was inoculated into 200 mL of LB medium supplemented with 100 mg/L ampicillin, followed by incubation at 37 °C with shaking. When the OD_600_ reached approximately 0.6, as measured using an ultraviolet spectrophotometer (NP80, Implen GmbH, Munich, Germany), IPTG was added to a final concentration of 1 mM to induce protein expression, with additional shaking at 16 °C for 16 h. Cells were harvested by centrifugation at 7000×*g* for 5 min at 4 °C (3-18 KS, Eppendorf, Hamburg, Germany) and then re-suspended in lysis buffer (20 mM Tris-HCl [pH 7.6], 300 mM KCl, 1 mM EDTA and 5 mM imidazole). After sonication (JY92-IIDN, Scientz, Ningbo, China) on ice for 45 min, samples were collected through centrifugation for 20 min at 15000×*g* and 4 °C. The supernatant was applied to a Ni-NTA affinity column pre-equilibrated with lysis buffer. To remove non-specifically bound proteins, the column was washed with wash buffer I (20 mM Tris-HCl [pH 7.6], 300 mM KCl and 40 mM imidazole) and wash buffer II (20 mM Tris-HCl [pH 7.6], 300 mM KCl and 80 mM imidazole). His-tagged fusion proteins were eluted with elution buffer (20 mM Tris-HCl [pH 7.6], 300 mM KCl and 250 mM imidazole). Eluted proteins were dialyzed against storage buffer (20 mM Tris-HCl [pH 7.6], 100 mM KC1, 0.5% (v/v) Tween 20, 0.5% (v/v) Nonidet P-40, 0.1 mM EDTA, 1 mM DTT, 50% glycerol) and stored at −20 °C. Protein concentrations were measured using the Bradford assay, with bovine serum albumin as the standard.

To optimize the enzymatic activity and storage stability of eMutS, we systematically evaluated the effects of ionic strength and buffer composition. The protein was dialyzed against buffers containing 50, 100, 150, or 200 mM KCl to identify the optimal potassium ion concentration for maximal activity. In parallel, the storage buffer was supplemented with conventional stabilizing agents (EDTA and DTT) as well as non-ionic detergents (Tween 20 and NP-40) to further enhance protein stability during storage.

### 2.5 Preparation of heteroduplex DNA containing various errors

To evaluate the binding specificity of all engineered fusion protein variants, 16 oligonucleotides were synthesized by Sangon Biotech Co. (Shanghai, China; Table S2). These oligonucleotides included twelve variants each containing a single-base substitution, one with a single-base deletion, one with a single-base insertion, and two fully complementary sequences to serve as perfect matches. Meanwhile, the different lengths of oligonucleotides were synthesized by Sangon Biotech Co (Shanghai, China; Table S2). and annealled to evaluate the binding efficiency of the protein. Through controlled annealing, these designed oligonucleotides were used to generate all relevant single-base mismatch types (e.g., G·T, A·C, etc.; Table S3), in addition to perfectly matched homoduplexes, via the formation of both heteroduplexes and homoduplexes (Table S3). Briefly, 10 μL of 10 × annealing buffer (100 mM Tris-HCl [pH 7.6], 500 mM NaCl and 10 mM EDTA) and 10 μL of the 10 mM oligonucleotides were added to a 0.2 mL PCR tube, with the final volume adjusted to 100 μL using nuclease-free water. The mixture was subjected to thermal cycling in a T100 thermocycler (Bio-Rad, California, United States) under the following program: initial denaturation at 95 °C for 5 min, followed by gradual reannealing through 699 cycles where the temperature decreased by 0.1 °C every 30 s. This gradual cooling promoted precise reannealing and heteroduplex formation. Simultaneously, complementary oligonucleotides were re-annealed to create homoduplex DNA using the aforementioned protocol.

### 2.6 Evaluation of fusion proteins binding specificity via bind-shift assay

To quantitatively assess the DNA binding affinity and mismatch selectivity of the eMutS variants, bind-shift assays were performed under varying protein-to-DNA molar ratios (ranging from 0:1 to 15:1). Each reaction contained purified fusion proteins and heteroduplex or homoduplex DNA, incubated for 10 min at room temperature. The mixtures were resolved on an 8% non-denaturing TBE PAGE gel at 130 V for 30 min. Nucleic acids were visualized using SYBR Gold staining. A distinct band shift-indicative of protein-DNA complex formation was observed for mismatch-containing heteroduplexes, while homoduplex DNA remained largely unbound and migrated freely. This result confirmed the specific affinity of fusion proteins for mismatched DNA and their capacity to selectively retain error-containing duplexes.

### 2.7 Assessment of error removal efficiency using eMutS mutants and streptavidin magnetic beads

The engineered eMutS fusion variants specifically recognize and bind to mismatched DNA sequences, facilitating the removal of synthetic errors. Streptavidin magnetic beads (Sangon Biotech Co.: D110557) effectively eliminate eMutS-biotin proteins that have undergone error-correction. The DNA mixture was prepared with several molar ratios of error-containing DNA and error-free DNA. The concentration of engineered eMutS fusion variants in the mixture was quantified using a fluorescence spectrophotometer (BioTek Synergy HT) by measuring fluorescence intensity at 488 nm excitation and an 507 nm emission [52]. This step ensured the determination of the optimal amount of streptavidin magnetic beads required for complete and cost-effective removal of eMutS-variants-DNA complexes.

Subsequently, error correction was performed as follows: (*i*) *Binding reaction*: the fusion protein and DNA were combined at an optimized molar ratio. An appropriate volume of 10 × binding buffer (50 mM MgCl_2_, 1 M KCl, 200 mM Tris-HCl [pH 7.6], 10 mM DTT) was added, and the total volume was adjusted to 30 μL with nuclease-free water. The mixture was incubated at room temperature for 10 min to facilitate specific binding of eMutS variants to mismatched DNA. (*ii*) *Magnetic bead capture*: the reaction mixture was transferred to a sterile tube containing pre-washed streptavidin magnetic beads. The sample was gently pipetted to ensure thorough mixing and incubated for 5 min at room temperature to allow complete capture of the protein-DNA complexes by the beads. (*iii*) *Separation and recovery*: the tube was placed on a magnetic rack for 5 min to immobilize the beads. The supernatant, containing error-corrected DNA, was carefully collected and subjected to high-throughput sequencing (Sangon Biotech Co.) to validate the error removal efficiency.

## 3 Results

### 3.1 Overview of the eMBS strategy for high-fidelity DNA synthesis error correction

**Fig. 1:**
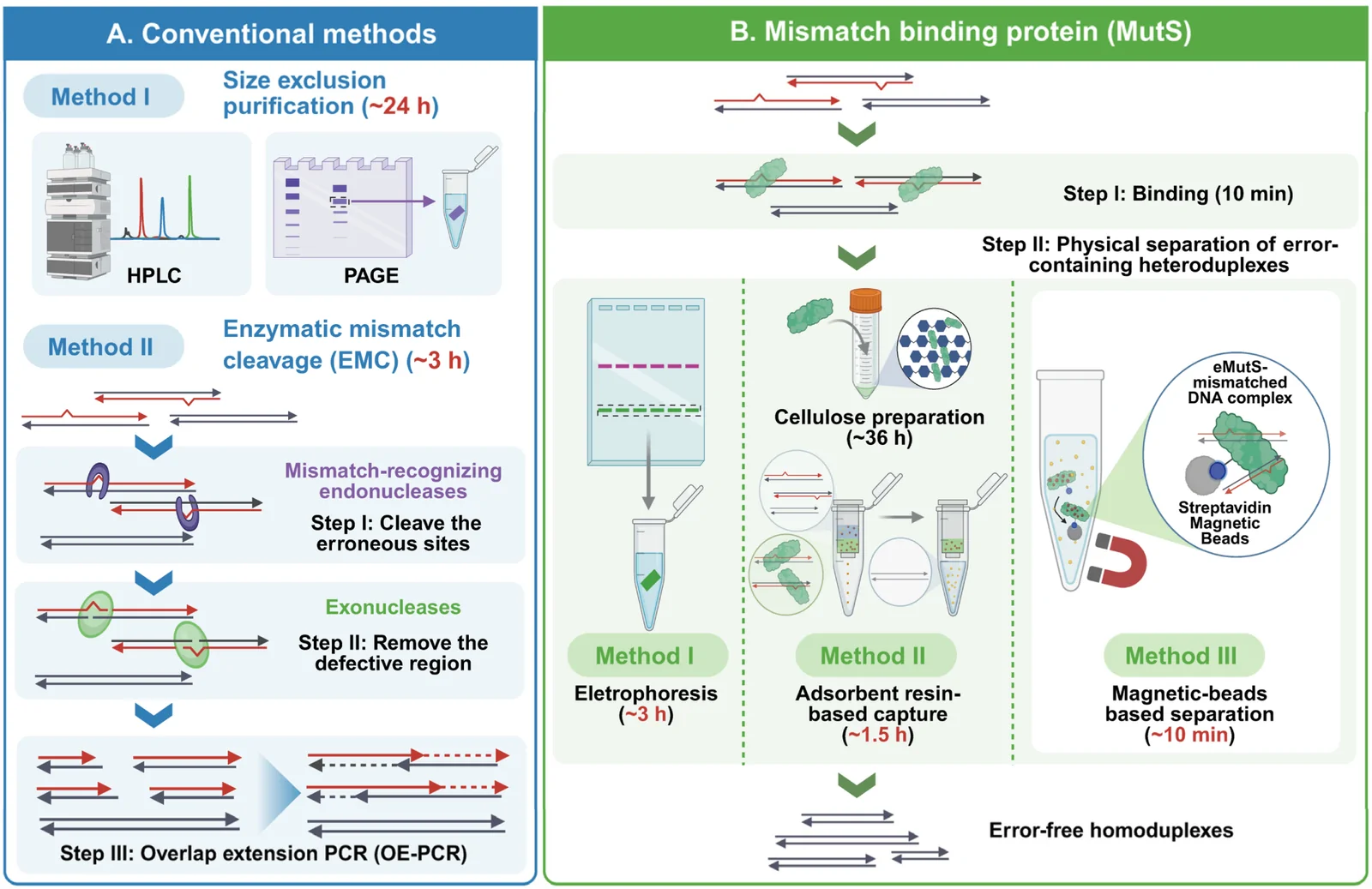
Strategies for error correction in DNA synthesis. (**A**) The schematic of conventional error-correction methods for DNA synthesis. (**B**) The schematic of mismatch binding protein (MutS) and its physical separation after error correction.

Conventional error-correction methods for DNA synthesis, such as enzymatic mismatch cleavage (EMC) systems (e.g., T7 endonuclease I or CorrectASE) and chromatography-based purification (e.g., HPLC or PAGE), are often constrained by multiple limitations: prolonged processing times (typically >24 h), labor-intensive and multi-step procedures (e.g., electrophoresis, column purification), limited compatibility with automation, and suffer from high reagent costs and variable specificity across different mismatch types. These factors collectively hinder their scalability and practicality for high-throughput gene synthesis (Fig. 1A).

**Fig. 2:**
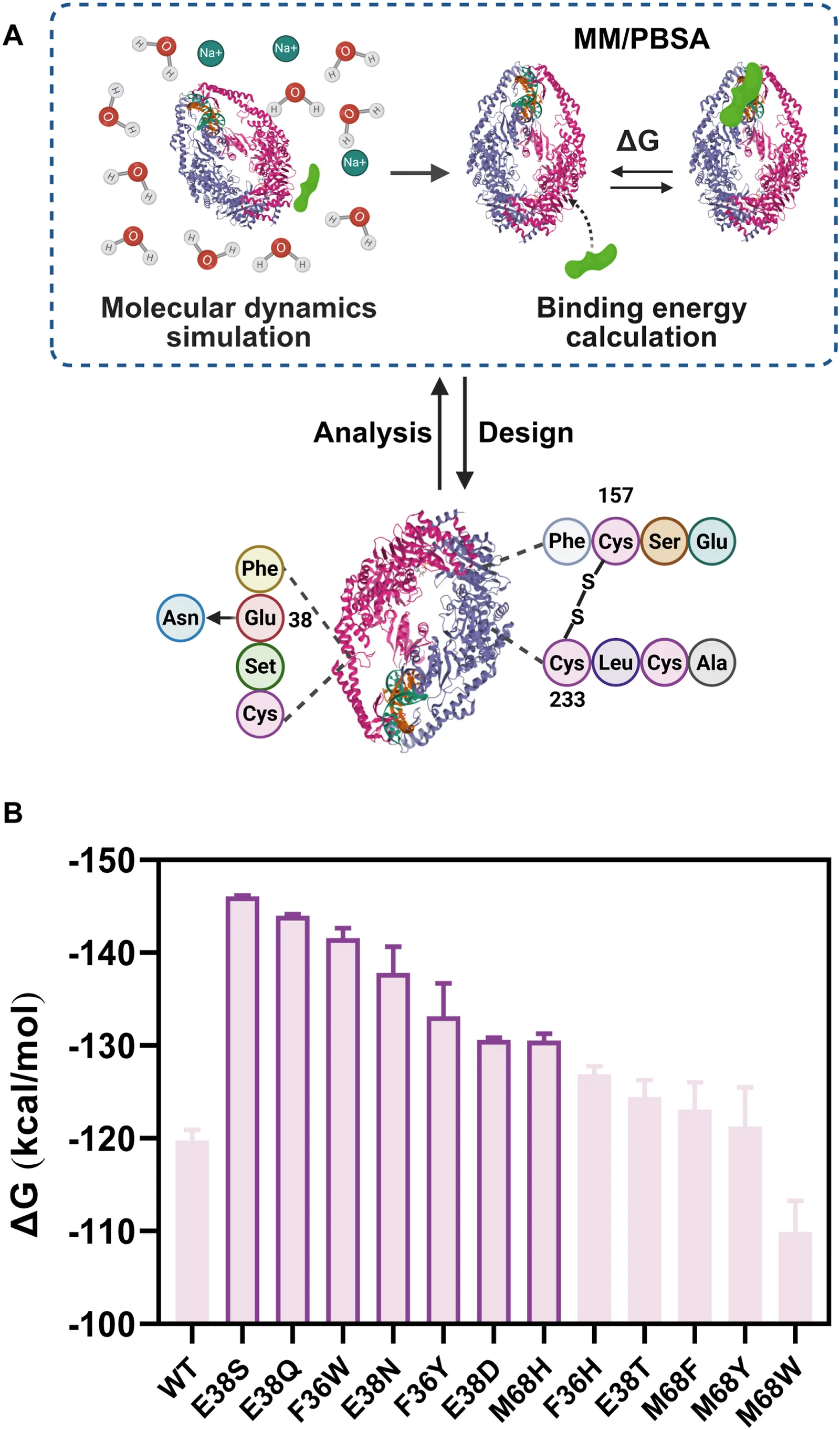
Rational design for the high specificity of eMutS protein. (**A**) The schematic of computational design and analysis of eMutS-L157C/G233C-biotin protein variants. (**B**) The binding free energies predicted by molecular dynamics (MD) simulations and MM/PBSA calculations.

In contrast, we developed an integrated, two-step error-correction platform, eMBS (engineered MutS-based magnetic bead system), that bypasses two inefficiencies through rational protein engineering, biophysical mechanism dissection, and streamlined automation-friendly workflows: (*i*) *Rational design of high-specificity eMutS variants*: we introduced a structure-guided strategy targeting residues critical for DNA recognition and binding. A point mutation (E38N) was designed to enhance mismatch specificity and reduce non-specific binding to perfectly matched DNA. Two additional cysteine mutations (L157C/G233C) were incorporated to promote disulfide bond formation, improving structural stability and affinity across all three error types (insertion, deletion and substitution). Molecular dynamics simulations and binding free energy calculations (MM/PBSA) confirmed that these mutations enhanced π–π stacking and hydrogen bonding with mismatched bases. The resulting variant, eMutS-L157C/G233C-E38N, was fused to EGFP and a biotin tag at the N-terminus for visualization and solid-phase immobilization (Fig. 2A). (*ii*) *System assembly and magnetic bead-based separation*: the purified eMutS-variant fusion protein was immobilized onto streptavidin-coated magnetic beads, forming the core of the eMBS. This design enables rapid and specific capture of error-containing heteroduplexes, and the entire process (from heteroduplex binding to error-free DNA recovery) is completed within 20 min and is fully compatible with standard liquid-handling robots (Fig. 1B). Unlike column-based systems (e.g., iMICC), which require manual packing, extensive washing, and elution, the magnetic bead-based workflow allows for one-step separation using a magnetic rack, reducing hands-on time and sample loss, outperforming previous eMutS variants and commercial kits.

### 3.2 Introduction of specific point mutation enhances the error-recognition specificity of eMutS-L157C/G233C-biotin

To enhance the error-recognition specificity of eMutS-L157C/G233C-biotin, we introduced structure-guided point mutations. Given that recognition accuracy for GT mismatches represents a critical performance metric, we focused on residues surrounding the mismatched thymine (T). In GT mispairs, only the T base protrudes into the protein's binding pocket, enabling direct interactions with surrounding amino acids. Based on the crystal structure of the eMutS-DNA complex (pdb: 1E3M), three key residues (F36/E38/M68) that directly interact with the mismatched thymine base (T) were selected as mutation sites. Residues F36 and M68 oriented perpendicularly to the thymine base plane, were mutated to histidine (H), phenylalanine (F), tyrosine (Y) and tryptophan (W) to modulate π-π stacking interactions. Residue E38, which lies parallel to the thymine (T) base plane, was substituted with aspartic acid (D), glutamine (Q), asparagine (N), threonine (T), and serine (S) to regulate its hydrogen bonding interaction with the base ring, thereby regulating the binding specificity of eMutS to DNA mismatch. The binding free energies (Δ*G*) of wild-type (WT) and all mutants were predicted by molecular dynamics (MD) simulations and MM/PBSA calculations (Fig. 2A). As shown in Fig. 2B, seven mutants with Δ*G* enhanced by more than 10 kcal/mol were selected as experimental candidates.

### 3.3 Recombinant expression and functional evaluation of eMutS-L157C/G233C-biotin protein variants

To evaluate the recombinant production and initial functional screening of engineered eMutS variants designed for enhanced mismatch recognition, five out of seven designed eMutS-L157C/G233C-biotin fusion proteins carrying single-point mutations were successfully overexpressed in *E. coli* BL21(DE3). Each construct exhibited robust soluble expression, as confirmed by EGFP fluorescence, enabling real-time localization and quantitative assessment. The mismatch-recognition capability of each variant was assessed using a 59 bp DNA heteroduplex mixture (43.8 ng/μL, containing defined mismatches) and a fully matched DNA homoduplex. Purified eMutS-L157C/G233C-biotin variants were incubated with these DNA duplexes at varying molar ratios of protein to DNA (0:1 to 15:1) for 10 min at 25 °C. This approach aimed to assess the selective binding of the engineered proteins to error-containing DNA. Since wild-type eMutS exhibits intrinsic affinity toward perfectly matched duplexes [30], it was crucial to evaluate the engineered variants for enhanced specificity toward heteroduplex DNA and reduced non-specific binding. And the optimal ratio of protein to DNA was determined based on two criteria: efficient removal of mismatch-containing heteroduplexes and minimal binding to error-free homoduplexes. Under native PAGE (8%), successful binding was indicated by the disappearance of free heteroduplex DNA, whereas the homoduplex control continued to migrate as free nucleic acid. This gel-shift strategy enabled quantitative comparison of the specificities and activities across all eMutS variants [3,9].

**Fig. 3:**
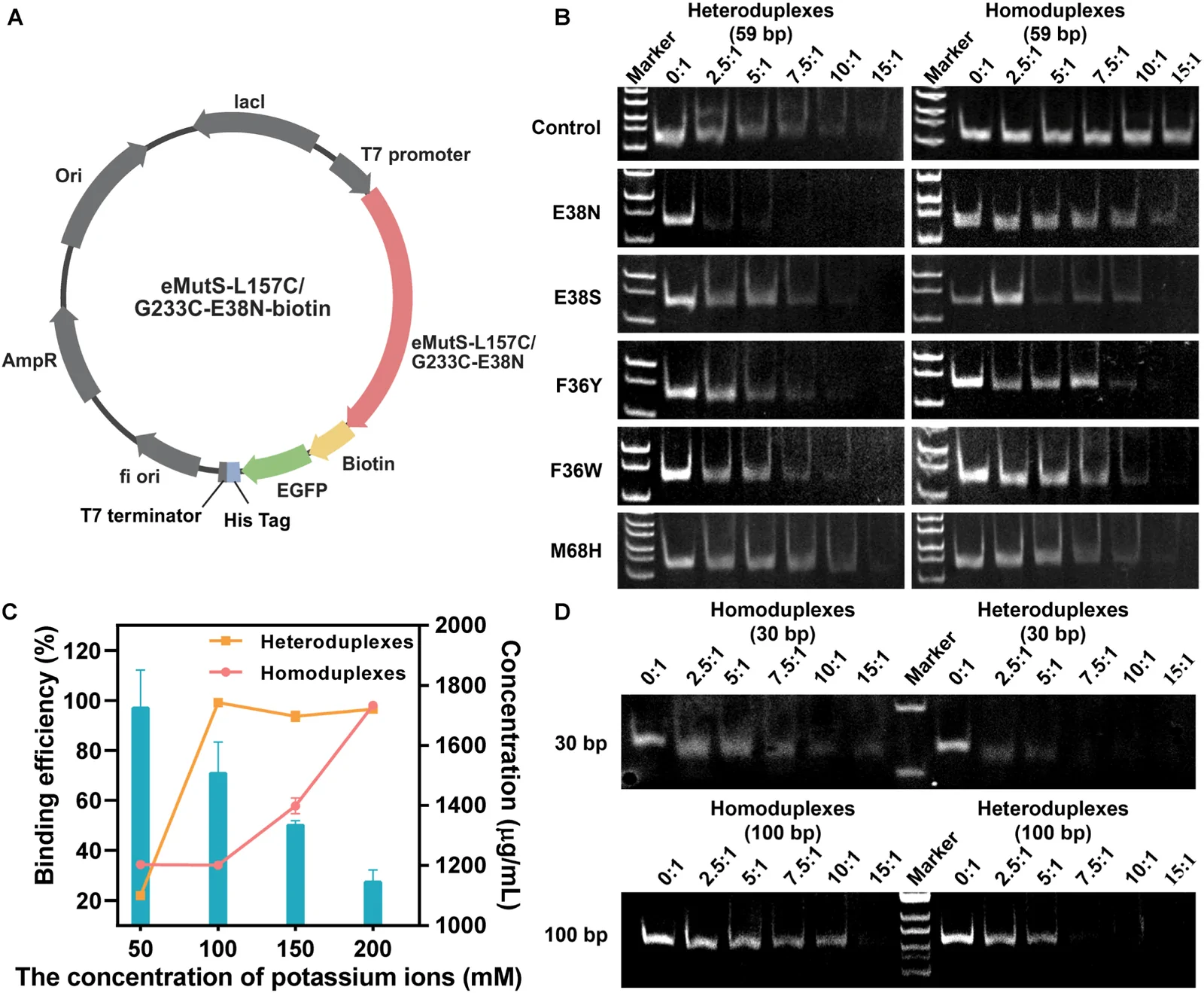
Band-shift assay of eMutS-L157C/G233C-biotin variants binding to heteroduplexes containing a single-base-pair mismatch and perfectly matched homoduplexes. (**A**) The expressed plasmid of eMutS-L157C/G233C-E38N-biotin. (**B**) The homoduplexes and heteroduplexes were incubated with various eMutS-biotin proteins across a range of molar ratios (protein:duplexes from 0:1 to 15:1) for 10 min at 25 °C. Control: eMutS-L157C/G233C-biotin protein. (**C**) The effects of potassium ion concentration (ranging from 50 to 200 mM) on protein concentration and eMutS-DNA binding efficiency. (**D**) The band-shift assays of eMutS with 30 bp and 100 bp lengths for heteroduplex or homoduplex DNA.

As shown in Fig. 3B, the eMutS-L157C/G233C-E38N-biotin mutant achieved near-complete binding of heteroduplex DNA at a protein-to-DNA ratio of 7.5:1, with minimal non-specific binding to the homoduplex. In contrast, the -F36W, -F36Y, and -E38S variants required a higher ratio of 15:1 to achieve comparable heteroduplex binding. Notably, the -M68H variant showed no detectable binding to mismatched DNA across all tested ratios (2.5:1 to 15:1) and failed to distinguish between heteroduplex and homoduplex substrates, rendering it unsuitable for error-correction applications. These findings underscore the functional importance of residues F36 and E38 in mediating mismatch recognition specificity. Based on these results, the eMutS-L157C/G233C-E38N-biotin, -F36W, -F36Y, and -E38S variants were selected for further evaluation using streptavidin magnetic bead-based separation assays with the unmutated eMutS-L157C/G233C-biotin construct included as a negative control.

Furthermore, to systematically evaluate the influence of storage buffer composition on both binding efficiency and enzymatic stability, we performed a comprehensive assessment of individual buffer components. Specifically, the effects of potassium ion concentration (50-200 mM) on protein concentration and eMutS-DNA binding efficiency were examined. As shown in Fig. 3C and Fig. S2, increasing potassium ion concentration was inversely correlated with the recovered protein concentration. In contrast, eMutS-DNA binding efficiency did not follow a monotonic trend across ionic strengths. Notably, storage in 100 mM KCl resulted in optimal binding discrimination, exhibiting the highest affinity toward error-containing DNA while maintaining minimal interaction with perfectly matched duplexes. In comparison, both low ionic strength (50 mM KCl) and higher potassium concentrations (≥150 mM KCl) led to suboptimal binding behavior.

In addition, to investigate the length dependence of mismatch recognition, DNA duplexes of 30 bp (26 ng/μL) and 100 bp (118 ng/μL) were designed and subjected to band-shift assays. As illustrated in Fig. 3D, eMutS-L157C/G233C-E38N-biotin exhibited negligible binding to 30 bp homoduplexes, while forming a weak complex with heteroduplexes, indicating limited but discernible mismatch discrimination at this length. Strikingly, when challenged with 100 bp substrates, the protein displayed highly selective binding to heteroduplex DNA at a protein-to-DNA molar ratio of 7.5:1, whereas only trace binding to homoduplex DNA was observed under identical conditions. Collectively, these results demonstrate that across different substrate lengths, the eMutS-L157C/G233C-E38N-biotin mutant retains strong specificity for heteroduplex DNA while exhibiting minimal non-specific binding to homoduplex DNA.

### 3.4 Error-correction efficiency of eMutS-L157C/G233C-biotin variants immobilized on streptavidin-coated magnetic beads

**Fig. 4:**
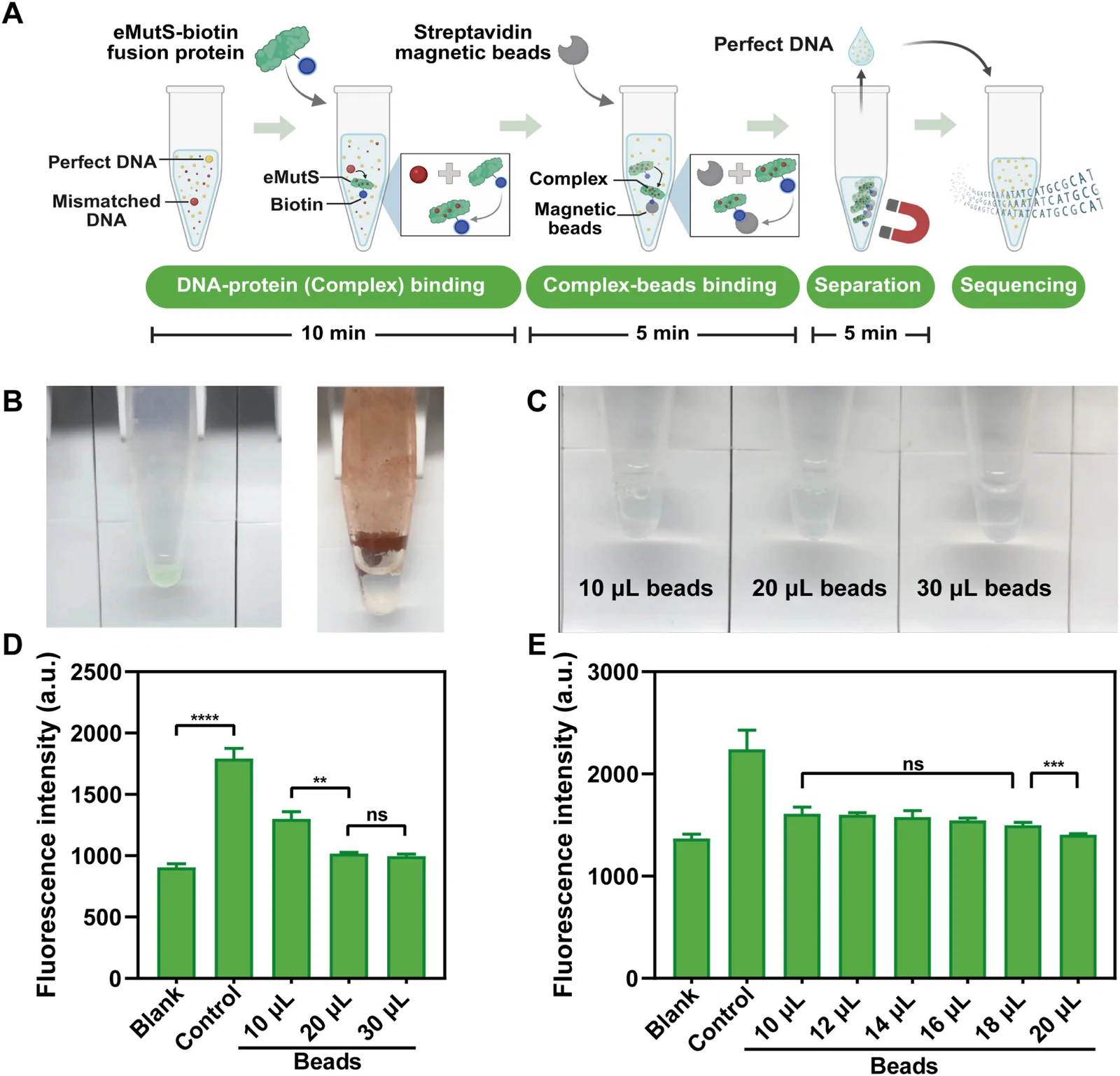
Evaluating the binding capacity of streptavidin magnetic beads for eMutS-L157C/G233C-biotin variants. (**A**) Schematic of the mismatch-removal system based on the eMutS-biotin-streptavidin magnetic bead platform. (**B**) Representative fluorescence images showing fusion protein before (left) and after (right) incubation with streptavidin magnetic beads. (**C**) Fluorescence signal of fusion protein following binding to streptavidin magnetic beads. (**D**) Quantification of fluorescence intensity in reaction solutions after incubation with increasing volumes of streptavidin magnetic beads (10 ∼30 μL, in 10 μL increments). (**E**) Fine-scale quantification of fluorescence intensity after binding with streptavidin magnetic beads across smaller volume intervals (10 ∼20 μL, in 2 μL increments).

To evaluate the error-removal capability of eMutS-L157C/G233C-biotin variants using streptavidin magnetic beads, we established an efficient and specific mismatch-removal system based on an eMutS-biotin-streptavidin magnetic bead platform (Fig. 4A). The process begins with a DNA sample containing both perfect and mismatch-containing heteroduplexes. The sample is first incubated with eMutS-biotin fusion protein, which selectively binds to mismatched DNA regions. The resulting protein-DNA complexes are then captured by streptavidin-coated magnetic beads. After magnetic separation, the supernatant (enriched with error-free homoduplex DNA) is collected for downstream sequencing, while the bead-bound complexes containing error-containing heteroduplexes are discarded. This efficient and specific capture system enables high-fidelity separation of synthetic DNA products (Fig. 4A).

The binding capacity of streptavidin magnetic beads for eMutS-L157C/G233C-biotin variants was assessed by measuring the fluorescence intensity of the supernatant, which directly corresponds to the amount of unbound fusion proteins remaining after bead capture (Fig. 4B–C). As shown in Fig. 4D, the control group (fusion protein without magnetic bead treatment) exhibited the highest fluorescence intensity at 1793 ± 82.52 a.u. (mean ± s.d., n = 3), representing the baseline signal. In contrast, the blank group (lacking fusion protein) demonstrated a significantly lower intensity of 906.0 ± 28.83 a.u. (*P* < 0.0001, two-tailed *t*-test), confirming the assay specificity. Using the positive correlation between eMutS concentration and fluorescence intensity, we titrated the streptavidin magnetic beads to determine the optimal amount. Specifically, increasing bead volumes from 10 to 30 μL at 10 μL intervals caused a progressive reduction in residual fluorescence to 1302 ± 58.28 a.u., 1018 ± 10.69 a.u., and 997 ± 16.46 a.u., respectively. Notably, the fluorescence intensity approached saturation between 20 and 30 μL, indicating near-complete capture of eMutS-biotin at 20 μL (Fig. 4D). Thus, we further refined this volume by testing 2 μL intervals from 10 μL to 20 μL (Fig. 4E). Fluorescence intensity remained statistically unchanged between 1498 ± 29.02 a.u. and 1610 ± 67.04 a.u. (one-way ANOVA, *P* = 0.0683), confirming that 20 μL was the optimal bead volume for a 30 μL incubation system. All error bars represent standard deviations from triplicate measurements. Thus, 20 μL of streptavidin magnetic beads (equivalent to 8 μg of streptavidin-coated particles) was selected for all subsequent experiments. Under these conditions, 1.5 μg/μL of fusion protein was quantitatively depleted from solution.

### 3.5 Assessment of error-correction efficacy of engineered various mutants

To evaluate the efficiency of engineered variants applied on the eMBS in selectively enriching error-free DNA from heterogeneous mixtures, DNA samples with initial error-free sequence proportions of 99.0% was subjected to mismatch removal followed by deep sequencing. As summarized in Table 1, all engineered variants reduced error rates to varying degrees across deletions, insertions, and substitutions-type errors, with the E38N mutant demonstrating the most substantial improvement.

**Table 1:** Comparison of error correction efficacy across engineered eMutS DNA variants.

Type of DNA		Before	After error correction				
			E38N	E38S	F36W	F36Y	M68H
**Perfect DNA**		78238	87881	112162	133591	120757	110391
**Deletion**		200	64	266	210	154	248
**Insertion**		181	62	194	142	87	218
**Substitution**		365	460	654	765	635	635
**Substitution**	**CT**	76	52	105	149	86	113
	**CA**	16	13	36	35	27	28
	**CG**	5	12	24	11	14	10
	**GT**	8	15	35	25	35	27
	**GA**	79	63	96	127	100	96
	**GC**	3	6	6	12	13	13
	**AC**	18	27	30	35	35	38
	**AG**	51	103	118	146	117	109
	**AT**	3	9	6	6	9	8
	**TC**	86	136	175	195	174	174
	**TG**	12	15	14	12	9	11
	**TA**	8	9	9	12	16	8
**Total error**		746	586	1114	1117	876	1101
**Bases sequenced**		78984	88467	113276	134708	121633	111492
**Ratio of error-free**		99.0%	99.3%	99.0%	99.1%	99.2%	99.0%

Abbreviations.

E38N: eMutS-L157C/G233C-E38N-biotin.

E38S: eMutS-L157C/G233C-E38S-biotin.

F36W: eMutS-L157C/G233C-F36W-biotin.

F36Y: eMutS-L157C/G233C-F36Y-biotin.

M68H: eMutS-L157C/G233C-M68H-biotin.

Specifically, *for deletion errors*, which initially appeared in 200 sequences, the E38N variant achieved a 68% reduction (from 200 to 64 errors). In comparison, the F36Y mutant reduced deletions by 23% (to 154 errors), while the other six mutants showed no significant improvement (Table 1). *Insertion errors*, initially totaling 181, were most effectively reduced by the E38N mutant (65.7% reduction, to 62 errors), followed by F36Y (51.9%, to 87 errors) and F36W (21.5%, to 142 errors). The remaining five mutants either failed to reduce insertion errors or slightly increased error rates (Table 1). *Substitution errors*, which include both transitions (e.g., G/C to A/T, A/T to G/C) and transversions (e.g., G/C to C/G, G/C to T/A, A/T to C/G, A/T to T/A), were also substantially decreased by the E38N variant. The most significant reductions were observed in the transitions, particularly in the GA and CT substitutions. For instance, the GA substitution errors in E38N decreased from 79 to 63 with a 20.3% reduction, and the CT substitution errors decreased from 76 to 52 with a 31.6% reduction. Similarly, transversions also showed notable decreases, with the CA substitution errors in E38N being reduced from 16 to 13 with an 18.8% reduction. Overall, the E38N mutant delivered the highest total error reduction, lowering the cumulative errors from 746 to 586 (21.4%). It also elevated the proportion of error-free bases to 99.3% in the initial 99.0% pool, outperforming the F36Y (99.2%) and F36W (99.1%) variants. Consistent with prior native PAGE analyses, E38N demonstrated minimal non-specific binding to error-free duplex DNA, as reflected by the lowest loss in total DNA yield among all variants.

In summary, the eMBS effectively enhances DNA sequence accuracy, with the E38N mutant demonstrating superior mismatch-binding specificity and the highest overall fidelity improvement. These findings highlight the potential of the E38N mutant for improving the accuracy of DNA sequencing applications.

### 3.6 Mismatch detection of the eMutS-L157C/G233C-E38N-biotin fusion protein via molecular dynamics simulations

**Fig. 5:**
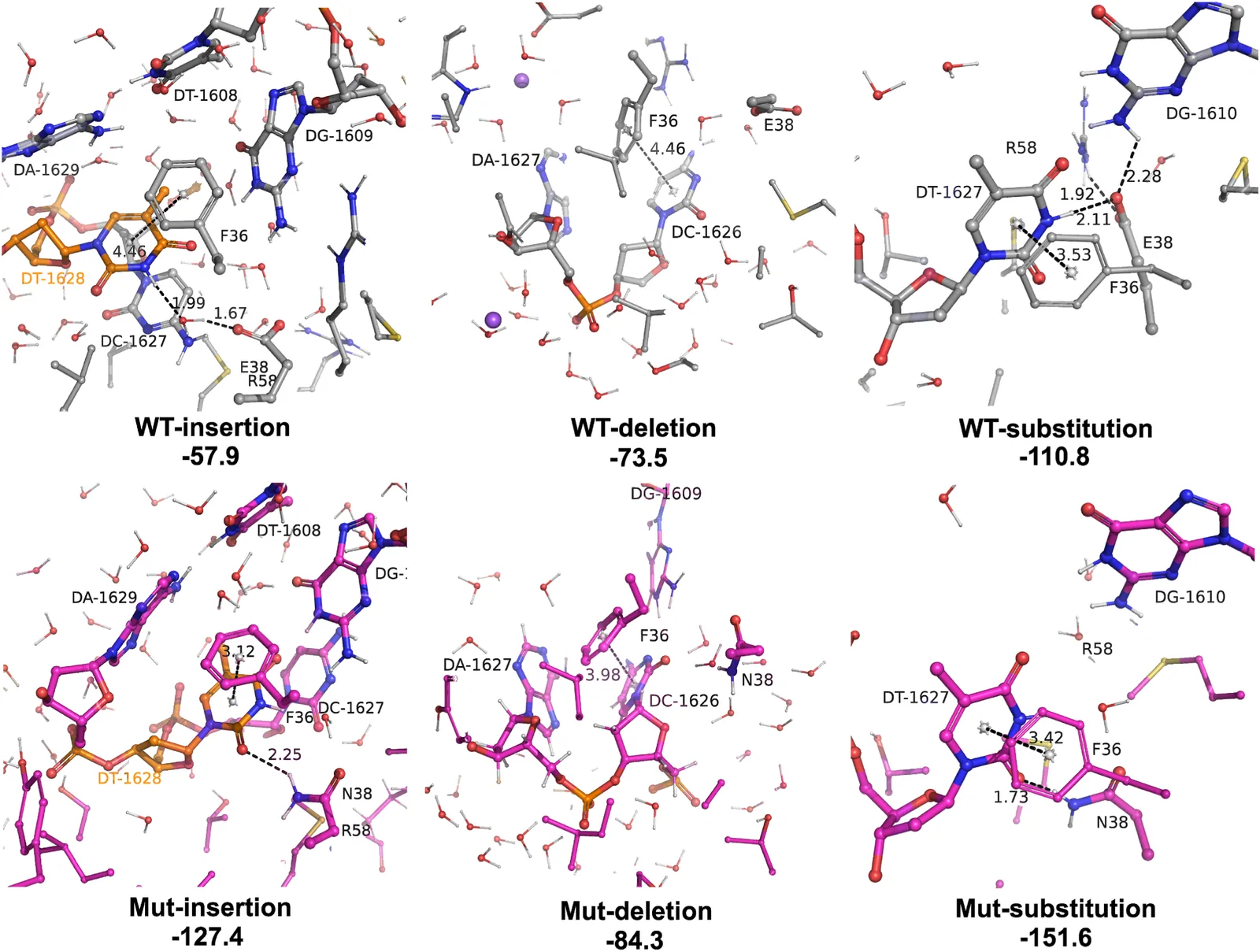
Comparison of binding affinities. Binding affinities of the wild-type (WT) enzyme and the L157C/G233C-E38N mutant (Mut) towards three error-containing DNA substrates (insertion, deletion, and substitution mismatches) as determined by molecular dynamics (MD) simulations and free energy calculations. Hydrogen bonds and key distances (in angstrom) are shown as dashed lines. The energies are given in kcal/mol.

Computational analysis was employed to quantify and compare the binding affinities of the wild-type (WT) eMutS and the engineered eMutS-L157C/G233C-E38N-biotin mutant towards three distinct error-containing DNA substrates (insertion, deletion, and substitution mismatches). Molecular dynamics (MD) simulations and free energy calculations revealed that the mutant variant exhibited significantly stronger binding affinity across all error types compared to the WT enzyme. This computationally derived enhancement in DNA mismatch recognition is consistent with prior experimental findings (Fig. 5).

A detailed structural analysis of the simulated complexes elucidated the mechanistic basis for the improved binding performance (Fig. 5). Specifically, for the insertion-error complex, the WT protein engaged the inserted thymine (T) via a water-mediated interaction with E38 and a weak π-π stacking through F36. In contrast, the mutant established a direct hydrogen bond with N38 and an enhanced π-π stacking through F36, which collectively boosted the affinity near the insertion site. In the deletion-error complex involving a T base, the mutation significantly strengthened the weak π-π stacking interaction observed in WT between F36 and C, thereby increasing binding affinity at the deletion site. In the substitution-error complex, both enzymes maintained π-π stacking between F36 and the mismatched T. However, a key distinction lies in the hydrogen bonding pattern: the WT E38 engaged in hydrogen bonds with both the template G and mismatched T, while the mutant N38 formed a singular, direct, and more stable hydrogen bond exclusively with the mismatched T.

Collectively, these findings demonstrate that the L157C/G233C-E38N mutations enhance binding affinity across all three error types by optimizing key residue-base interactions, specifically through strengthened π-π stacking and the formation of direct hydrogen bonds.

### 3.7 Engineered eMBS enables robust DNA error correction across diverse substrate fidelities

**Fig. 6:**
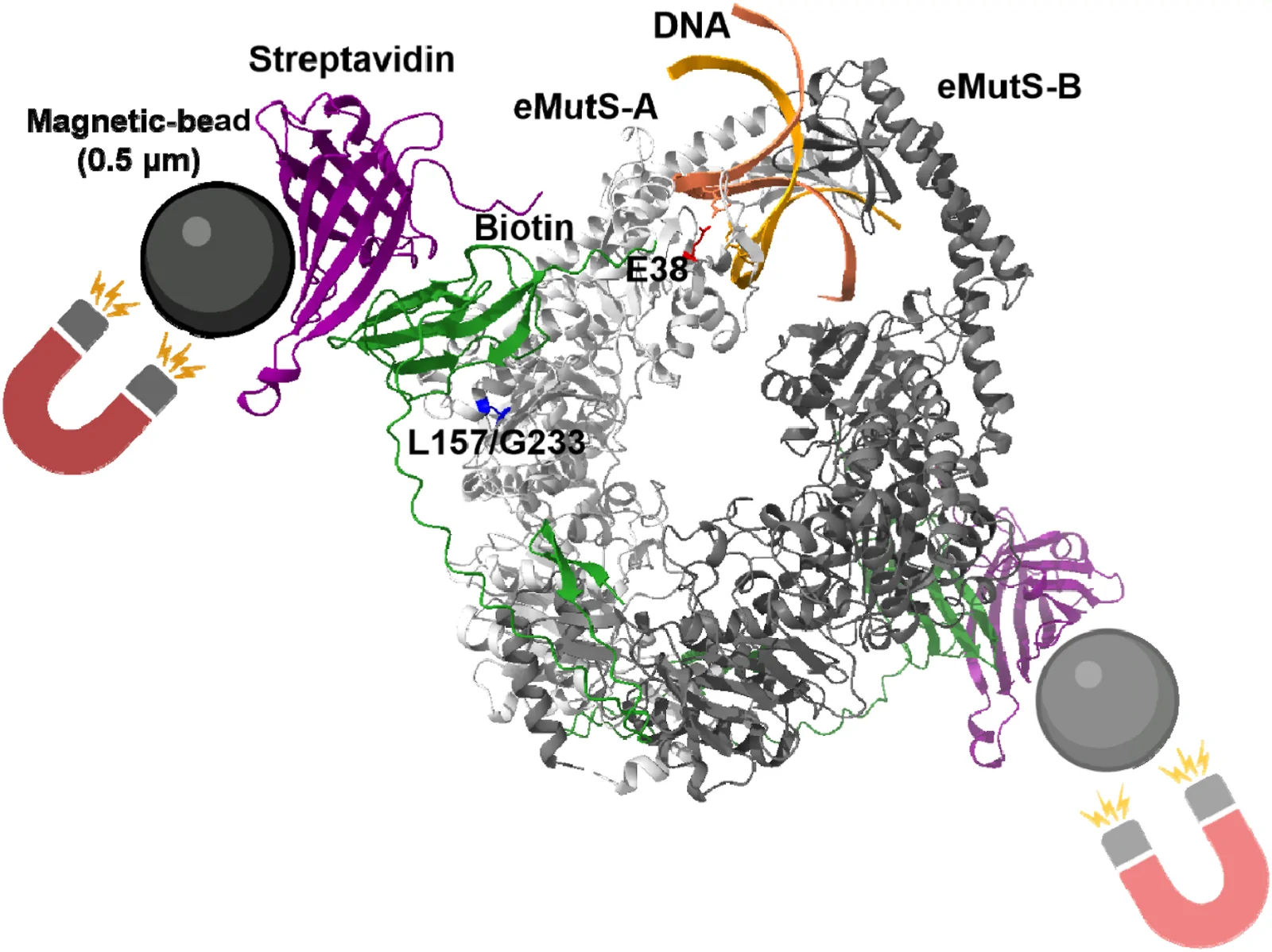
The structure of eMBS. Grey: eMutS; Red: single point mutant (E38N); Blue: disulfide bond (L157C/G233C); Green: biotin (predicted by Alphafold 3); Purple: streptavidin (predicted by Alphafold 3); Yellow: DNA; Black: magnetic-bead (diameter: 0.5 μm).

A comprehensive evaluation of the error-correction efficiency was conducted using two engineered enzymes: eMutS-L157C/G233C-biotin protein and its E38N mutant, across DNA substrate pools with varying initial fidelities (73.6%, 92.8%, and 98.6%). The eMBS incorporates a single-point mutant (E38N) coupled with a disulfide bond (L157C/G233C) and biotin as interaction with streptavidin magnetic-beads (Fig. 6), and consistently demonstrated superior performance in enhancing sequencing accuracy and specificity across all error types and initial fidelity levels.

Using a low-fidelity substrate with an initial error-free fraction of 73.6% (87,957 perfect bases), both variants significantly improved accuracy. The eMutS-L157C/G233C-biotin increased the number of error-free bases to 105,583, while its E38N mutant achieved a further improvement to 107,849, demonstrating its superior corrective capability (Table 2). And the initial error rate of 26.4% (31,521 errors) was reduced to 12.2% (14,727 errors) by the eMutS-L157C/G233C-biotin, reflecting a 1.16-fold enhancement in accuracy. In comparison, the eMutS-L157C/G233C-E38N-biotin further suppressed the error count to 9.7% (11,567 errors), achieving a 1.26-fold improvement over the parent protein and a 2.72-fold gain compared to the uncorrected control. Consequently, the final error-free ratios rose to 87.8% and 90.3%, respectively (Table 2).

**Table 2:** Comparison of the error-correction efficiency between the eMutS-L157C/G233C-biotin protein and its E38 N mutant^a^.

Type of DNA		73.6%			92.8%			98.6%		
		Before	After		Before	After		Before	After	
			eMutS	E38N		eMutS	E38N		eMutS	E38N
Perfect DNA		87957	105583	107849	83625	80414	79471	65685	81644	86961
Deletion		13130	5075	4194	2778	1529	1174	340	306	280
Insertion		8700	2924	1772	1649	373	447	169	74	58
Substitution		9691	6728	5601	2021	1399	1354	437	506	484
Substitution	CT	1410	697	543	351	127	156	68	53	50
	CA	975	559	365	169	116	135	14	17	17
	CG	421	356	467	72	66	51	7	10	11
	GT	1034	598	336	201	121	131	21	34	19
	GA	1046	603	379	224	143	156	88	85	74
	GC	460	368	346	80	53	57	12	19	19
	AC	916	551	377	178	153	119	23	36	35
	AG	777	592	408	188	161	159	57	75	69
	AT	449	430	601	65	66	80	15	14	15
	TC	1048	883	787	290	234	161	99	129	138
	TG	675	558	387	136	87	89	22	15	20
	TA	480	533	605	67	72	60	11	19	17
Total error		31521	14727	11567	6448	3301	2975	946	886	822
Bases sequenced		119478	120310	119416	90073	83715	82446	66631	82530	87783
Ratio of error-free		73.6%	87.8%	90.3%	92.8%	96.1%	96.4%	98.6%	98.9%	99.1%

Abbreviations.

eMutS: eMutS-L157C/G233C-biotin.

E38N: eMutS-L157C/G233C-E38N-biotin.

a Data shown are from three independent correction experiments (n = 3).

A detailed breakdown by error type revealed that both enzymes exhibited a strong preference for resolving insertion and deletion errors, with the eMutS-L157C/G233C-E38N-biotin displaying superior specificity. Deletion errors were reduced from 13,130 to 5075 (61.3% decrease) using the eMutS-L157C/G233C-biotin, and further to 4194 (68.0% decrease) with its E38N mutant. Insertions declined from 8700 to 2924 (66.4% reduction) by the eMutS-L157C/G233C-biotin and 1772 (79.6% reduction) by its E38N mutant, respectively. The eMutS-L157C/G233C-E38N-biotin also showed improved suppression of substitution errors, notably reducing prevalent transversions such as C/T from 1410 to 543 (a 22% greater reduction than achieved by the eMutS-L157C/G233C-biotin). Similar enhancements were observed across other substitution types (C/A, G/T, G/A, G/C, A/C, A/G, T/C and T/G), underscoring the broader error-resolution capacity conferred by the eMutS-L157C/G233C-E38N-biotin (Table 2).

The proteins were further evaluated on higher-fidelity substrates with initial accuracies of 92.8% and 98.6%. For the 92.8% pool, the eMutS-L157C/G233C-biotin elevated the error-free rate to 96.1%, while eMutS-L157C/G233C-E38N-biotin reached 96.4%. In the 98.6% pool, eMutS-L157C/G233C-biotin maintained baseline accuracy, whereas eMutS-L157C/G233C-E38N-biotin achieved a slight improvement to 99.1% (Table 2). Total error-correction efficiency was quantified across both pools. In the 92.8%-fidelity substrate (initial errors = 6448), the eMutS-L157C/G233C-biotin and eMutS-L157C/G233C-E38N-biotin reduced error counts to 3301 (48.8% efficiency) and 2840 (53.9% efficiency), respectively. For the 98.6%-fidelity substrate (initial errors = 946), eMutS-L157C/G233C-biotin achieved a 6.3% reduction (to 886 errors), while eMBS reached 13.1% (822 errors; Table 2).

Error-specific analysis within the 92.8% pool showed that the eMutS-L157C/G233C-E38N-biotin corrected deletion errors more effectively (57.8% vs. 44.8% achieved by eMutS-L157C/G233C-biotin), and also outperformed eMutS-L157C/G233C-biotin in addressing specific substitution types such as C/G, A/C, A/G, and T/C (from 72, 178, 188, 290 to 66, 153, 161, 234; Table 2). In the 98.6% pool, the eMutS-L157C/G233C-E38N-biotin reduced deletions by 17.6% (from 340 to 280), compared to 10.0% (from 340 to 306) by eMutS-L157C/G233C-biotin, and insertions by 65.7% (from 169 to 58), exceeding eMutS-L157C/G233C-biotin's 56.8% (from 169 to 74). While some substitution classes exhibited modest gains, the eMutS-L157C/G233C-E38N-biotin consistently matched or exceeded the performance of eMutS-L157C/G233C-biotin construct across most error types (Table 2).

To further extend the application of eMBS, its error-correction efficiency was evaluated using longer DNA substrate pools (100 bp). In the pool with an initial error-free fraction of 70.4%, the number of error-free bases increased from 39,970 to 40,928, whereas in the 87.0% pool, this value increased from 54,773 to 56,655. Correspondingly, the initial error rates of 29.6% and 13.0% were reduced to 21.8% and 6.2%, respectively, representing an approximately twofold improvement in accuracy. As a result, the final error-free fractions increased to 78.2% and 93.8%, respectively (Table S4). A detailed error-type analysis revealed that eMBS displayed a pronounced preference for correcting insertion and deletion errors. Specifically, deletion errors decreased from 7653 to 5946 (22.3% reduction) in the 70.4% pool and from 3786 to 2034 (46.3% reduction) in the 87.0% pool. Similarly, insertion errors declined from 7682 to 4268 (44.4% reduction) and from 3856 to 1121 (70.9% reduction) in the respective pools. Comparable improvements were also observed for substitution errors, which were reduced by 19.0% (from 1443 to 1169) and 42.8% (from 820 to 469) in the two DNA pools. Collectively, these results underscore the broad and effective error-resolution capacity conferred by eMBS when applied to longer DNA substrate pools.

In summary, the eMBS strategy significantly enhances DNA sequencing accuracy through robust mismatch recognition and error removal, with the E38N point mutation conferring consistently superior performance, particularly for deletions, insertions, and several substitution types. These results highlight the value of the eMBS strategy in applications demanding ultra-high-fidelity DNA synthesis and support its integration into scalable, high-throughput error-correction workflows.

## 4 Discussion

The pursuit of high-fidelity DNA synthesis is fundamental for advancing synthetic biology, particularly in the reliable construction of long DNA sequences and artificial genomes [5]. In this study, we systematically enhanced the error-correction performance of eMutS through three integrated strategies (*i*) rational redesign of eMutS to improve mismatch recognition specificity and binding affinity for erroneous DNA while minimizing non-specific binding to correct duplexes; (*ii*) multiscale theoretical simulations to dissect the MutS mismatch identification mechanism at atomic resolution, identifying key residues and structural motifs governing fidelity; and (*iii*) development of a magnetic bead-based workflow that eliminates column-based recovery and minimizes hands-on time. Our findings demonstrate that rational protein engineering combined with magnetic bead immobilization, named eMBS strategy, significantly facilitates the practical gene synthesis applications by improving both specificity and compatibility with automated workflows.

Enzymatic error correction remains the most effective method for rectifying errors in synthetic DNA (Fig. 1A). However, the limited availability and high cost of commercial reagents (such as Surveyor, CorrectASE, and PreCR, which are proprietary enzyme mixtures leveraging multiple activities for enhanced correction) pose significant challenges for large-scale synthetic biology applications [22,53]. To overcome these limitations, we engineered eMutS to improve its specificity and automation compatibility. By integrating computational binding free energy calculations into rational design, we significantly improved the DNA mismatch recognition specificity of eMutS. Rational design strategies (particularly those guided by molecular dynamics simulations) effectively mitigate the drawbacks of irrational design, which often relies on large mutant libraries and labor-intensive screening in the absence of efficient high-throughput methods [54,55]. Our results confirm that this approach markedly enhances enzyme binding specificity. Specifically, the introduction of the E38N mutation into eMutS-L157C/G233C-biotin substantially improved its ability to discriminate between error-containing and error-free DNA. Unlike the parent protein, which exhibited nonspecific binding to both DNA types, the E38N variant achieved near-complete mismatch removal at a protein-to-DNA molar ratio of 7.5:1 while effectively preserving perfectly matched DNA. This specificity minimizes the loss of error-free DNA during correction and is attributed to the L157C/G233C-E38N mutations, which enhance binding affinity for all three error types via optimized key residue-base interactions, specifically through strengthened π-π stacking and the formation of direct hydrogen bonds.

Transitioning from protein design to application, we contextualize our eMBS strategy within the broader workflow of MutS-mediated correction. In a conventional MutS-based error correction workflow (Fig. 1B), synthetic DNA fragments are denatured and reannealed to form heteroduplexes, thereby exposing synthesis errors (e.g., mismatches and indels) as non-complementary base pairs. MutS selectively recognizes these aberrant structures, enabling the physical separation of error-containing heteroduplexes from error-free homoduplexes. While downstream purification has traditionally relied on gel electrophoresis, capillary electrophoresis, affinity beads, or adsorbent resins (all involving considerable operational complexity), our integrated eMBS strategy overcomes these limitations by simultaneously enhancing specificity and automation compatibility. Several magnetic-bead platforms depleted oligonucleotides bearing deletions or insertions, but they consistently failed to enrich sequences containing single-base substitutions [56,57]. Owing to the differential binding affinity of our integrated eMBS strategy towards various error types, the eMutS-L157C/G233C-E38N-biotin mutant demonstrated robust error-correction performance across DNA substrate pools with varying initial fidelities (73.6%, 92.8%, and 98.6%). Sequencing results showed that eMBS reduced total errors by 13.1∼63.3% in pools initially containing 73.6∼98.6% correct sequences, and achieved an error-free DNA ratio of 99.1% which is significantly outperforming of iMICC (90.4%). The E38N variant selectively eliminated insertion and substitution errors, reducing their frequencies by 79.6% and 42.2% in the 73.6% pool, and by 72.9% and 33.0% in the 92.8% pool (Table 2), respectively. These findings indicate a pronounced preference for T-base mismatches (both inserted and substituted), aligning with the original design objective and mismatch identification mechanism studies. Additionally, the removal efficiencies for CT and GT substitution errors declined gradually with increasing substrate fidelity (CT: 61.5%, 55.6%, 26.5%; GT: 67.5%, 34.8%, 9.5%). Although deletion errors also exhibited substrate-dependent efficiency, the E38N mutant consistently outperformed the parental eMutS-L157C/G233C-biotin across all fidelity levels (68.1% vs. 61.3%, 57.7% vs. 45%, and 17.6% vs. 10%). While the eMBS demonstrates high efficiency in reducing overall error rates, it is important to note that its correction efficacy can vary depending on the mismatch type. For instance, the binding affinity and subsequent removal efficiency for single-base substitutions (e.g., C:G) may differ from that for insertion/deletion (e.g., +T). Collectively, these results demonstrate that the eMBS significantly enhances deletion error removal universally and substantially improves correction of insertion and substitution errors in lower-fidelity substrates, particularly those involving T-base mismatches.

Furthermore, eMBS offers significantly higher throughput and automation compatibility. By integrating a biotin-streptavidin magnetic bead system, it eliminates labor-intensive electrophoresis (PAGE) and column purification steps (iMICC), enabling seamless compatibility with standard liquid-handling platforms and high-throughput DNA synthesis workflows. Specifically, eMBS method completes the process in just 20 min, far faster than PAGE (24 h), EMC (3 h), and iMICC (1.5 h). Additionally, eMBS accommodates a broader DNA target range of 30-100 bp, enabling the processing of shorter fragments compared to other methods. Most notably, eMBS achieves an exceptional throughput of 96 tubes per single operation, dramatically outperforming alternative approaches in both speed and yield (Table 3). This scalability is critical for modern synthetic biology applications, as evidenced by numerous academic and industrial efforts (such as iBioFAB [58], the Codex DNA Gibson assembly platform [59], Ginkgo Bioworks, the Edinburgh Genome Foundry [60], the Tianjin Institute of Industrial Biotechnology [61], and the Shenzhen Synthetic Biology Research Center [62]) that are actively developing automated DNA assembly systems.

**Table 3:** Comparison of the separation methods about the utilization of the mismatch binding protein.

	Consuming time	DNA target	Throughput
**PAGE**	24 h	≥60 bp	10 gels/single operation
**EMC**	3 h	100-900 bp	one tube/single operation
**iMICC**	1.5 h	63-115 bp	one tube/single operation
**eMBS**	20 min	30-100 bp	96 tubes/single operation

Looking forward, while our engineered eMBS demonstrates broad specificity across various single-base substitutions, insertions, and deletions, its correction efficiency varies depending on the types of DNA mismatch. For instance, +T insertions were corrected more effectively than C:G substitutions. Future work will leverage molecular dynamics simulations to design additional mutations to expand mismatch recognition [33,37]. Although this study was applied in short DNA fragments rather than in large scale DNA synthesis, extending the system to longer constructs represents a key objective of our subsequent research. Simultaneously, scaling this technology for kilobase-scale DNA synthesis will necessitate the integration of eMBS with other mismatch-binding enzymes, such as Endonuclease V or T7 Endonuclease I, to address errors not efficiently recognized by MutS [20,22]. Furthermore, future explorations could assess the potential of engineered eMutS variants in recognizing mismatches in non-canonical nucleic acid structures, such as DNA-RNA hybrids, which might open up new avenues for molecular diagnostics. Such integration is expected to further enhance the robustness and applicability of our error-correction system, thereby paving the way for high-fidelity DNA synthesis in various synthetic biology applications.

## 5 Conclusions

Our study establishes a robust framework for error correction in DNA synthesis by integrating rational enzyme engineering with scalable immobilization strategies. The eMBS achieved an error-free DNA ratio of 99.1% while significantly reducing labor-intensive steps, effectively addressing major bottlenecks in high-throughput synthetic biology workflows. Furthermore, molecular dynamics simulations and free energy calculations provided atomistic insights into the MutS mismatch identification mechanism, thereby providing deeper insights into its mismatch recognition specificity. As the demand for high-fidelity DNA continues to grow (spanning genome-scale engineering and DNA data storage applications), our technology offers a versatile and efficient foundation for next-generation error-correction tools. By providing high accuracy and seamless integration with automated systems, this approach holds promise for future applications in CRISPR-based genome editing, molecular diagnostics, and other fields where nucleic acid precision is critical.

## CRediT authorship contribution statement

**Xiaohang Wang:** Writing – original draft, Methodology, Investigation, Data curation. **Yefei Wang:** Writing – original draft, Funding acquisition, Data curation. **Wei Wang:** Writing – original draft, Data curation. **Wenshuang Liu:** Data curation. **Hui Jia:** Data curation. **Mengsha Li:** Visualization. **Mingying Li:** Data curation. **Changle Wu:** Formal analysis. **Jian Xu:** Resources. **Xiang Sheng:** Writing – review & editing, Supervision, Project administration, Funding acquisition. **Jia Zhang:** Writing – review & editing, Supervision, Project administration, Funding acquisition.

## Declaration of competing interest

The authors declare that they have no known competing financial interests or personal relationships that could have appeared to influence the work reported in this paper.
