# Supplementary material for: Engineered eMutS based magnetic separation system for high-fidelity DNA synthesis
Source: Synth Syst Biotechnol. 2026 Mar 6;13:337–49. doi: 10.1016/j.synbio.2026.02.009 (PMC13494400; doi:10.1016/j.synbio.2026.02.009)
Supplement: Supplementary file 1 [file mmc1.docx]

**Supplemental Tables and Figures**

**Table S1. The plasmids used in this study.**

| **Plasmids** | **Selection marker** | **Reference** |
| --- | --- | --- |
| peMutS-L157C/G233C-CBM3-EGFP | AMP | ^1^ |
| peMutS-L157C/G233C-EGFP-biotin | AMP | This study |
| peMutS-L157C/G233C-E38N-biotin | AMP | This study |
| peMutS-L157C/G233C-E38S-biotin | AMP | This study |
| peMutS-L157C/G233C-E38D-biotin | AMP | This study |
| peMutS-L157C/G233C-E38Q-biotin | AMP | This study |
| peMutS-L157C/G233C-F36W-biotin | AMP | This study |
| peMutS-L157C/G233C-F36Y-biotin | AMP | This study |
| peMutS-L157C/G233C-M68H-biotin | AMP | This study |

**Table S2. List of oligos synthesized for heteroduplex or homoduplex DNA construction for assaying the enzymatic activity of proteins.**

| **Oligos names** | **Oligos sequences** |
| --- | --- |
| Mutsgene-F | GCGGACTATTTAACACAGCTTTAGGCGCTGGACGAGGTACTATGAATCGGCCTTGCTCC |
| Mutsgene-R | CGCCTGATAAATTGTGTCGAAATCCGCGACCTGCTCCATGATACTTAGCCGGAACGAGG |
| Mutsgene-R-QS | CGCCTGATAAATTGTGTCGAAATCCGCGACTGCTCCATGATACTTAGCCGGAACGAGG |
| Mutsgene-R-CR | CGCCTGATAAATTGTGTCGAAATCCGCGATCCTGCTCCATGATACTTAGCCGGAACGAGG |
| Mutsgene-R-TH-CT | CGCCTGATAAATTGTGTCGAAATCCGCGATCTGCTCCATGATACTTAGCCGGAACGAGG |
| Mutsgene-R-TH-CA | CGCCTGATAAATTGTGTCGAAATCCGCGAACTGCTCCATGATACTTAGCCGGAACGAGG |
| Mutsgene-R-TH-CG | CGCCTGATAAATTGTGTCGAAATCCGCGAGCTGCTCCATGATACTTAGCCGGAACGAGG |
| Mutsgene-R-TH-GT | CGCCTGATAAATTGTGTCGAAATCCGCTACCTGCTCCATGATACTTAGCCGGAACGAGG |
| Mutsgene-R-TH-GA | CGCCTGATAAATTGTGTCGAAATCCGCAACCTGCTCCATGATACTTAGCCGGAACGAGG |
| Mutsgene-R-TH-GC | CGCCTGATAAATTGTGTCGAAATCCGCCACCTGCTCCATGATACTTAGCCGGAACGAGG |
| Mutsgene-R-TH-AC | CGCCTGATAAATTGTGTCGAAATCCGCGCCCTGCTCCATGATACTTAGCCGGAACGAGG |
| Mutsgene-R-TH-AG | CGCCTGATAAATTGTGTCGAAATCCGCGGCCTGCTCCATGATACTTAGCCGGAACGAGG |
| Mutsgene-R-TH-AT | CGCCTGATAAATTGTGTCGAAATCCGCGTCCTGCTCCATGATACTTAGCCGGAACGAGG |
| Mutsgene-R-TH-TC | CGCCTGATAAATTGTGTCGAAATCCGCGACCCGCTCCATGATACTTAGCCGGAACGAGG |
| Mutsgene-R-TH-TG | CGCCTGATAAATTGTGTCGAAATCCGCGACCGGCTCCATGATACTTAGCCGGAACGAGG |
| Mutsgene-R-TH-TA | CGCCTGATAAATTGTGTCGAAATCCGCGACCAGCTCCATGATACTTAGCCGGAACGAGG |
| gene30-F | CAGCTTTAGGCGCTGGACGAGGTACTATGA |
| gene30-R | TCATAGTACCTCGTCCAGCGCCTAAAGCTG |
| gene30-R-QS | TCATAGTACCTCGTCAGCGCCTAAAGCTG |
| gene30-R-CR | TCATAGTACCTCGTCCTAGCGCCTAAAGCTG |
| gene30-R-TH | TCATAGTACCTCGTCTAGCGCCTAAAGCTG |
| gene100-F | ACGGATCCGCTAGCAGGATCCACTAGTATCGATCAGCTTTAGGCGCTGGACGAGGTACTATGACGATCGACTGCAGAATTCGAGCTCGATATCGAATTCG |
| gene100-R | CGAATTCGATATCGAGCTCGAATTCTGCAGTCGATCGTCATAGTACCTCGTCCAGCGCCTAAAGCTGATCGATACTAGTGGATCCTGCTAGCGGATCCGT |
| gene100-R-QS | CGAATTCGATATCGAGCTCGAATTCTGCAGTCGATCGTCATAGTACCTCGTCAGCGCCTAAAGCTGATCGATACTAGTGGATCCTGCTAGCGGATCCGT |
| gene100-R-CR | CGAATTCGATATCGAGCTCGAATTCTGCAGTCGATCGTCATAGTACCTCGTCCTAGCGCCTAAAGCTGATCGATACTAGTGGATCCTGCTAGCGGATCCGT |
| gene100-R-TH | CGAATTCGATATCGAGCTCGAATTCTGCAGTCGATCGTCATAGTACCTCGTCTAGCGCCTAAAGCTGATCGATACTAGTGGATCCTGCTAGCGGATCCGT |

**Table S3. List of synthesized double-stranded DNA samples for assaying the enzymatic activity of proteins.**

| **Double-stranded DNA name** | **Single-stranded DNA name** |
| --- | --- |
| Perfect DNA | Mutsgene-F and Mutsgene-R |
| Deletion | Mutsgene-F and Mutsgene-R-QS |
| Insertion | Mutsgene-F and Mutsgene-R-CR |
| Substitution-CT | Mutsgene-F and Mutsgene-R-TH-CT |
| Substitution-CA | Mutsgene-F and Mutsgene-R-TH-CA |
| Substitution-CG | Mutsgene-F and Mutsgene-R-TH-CG |
| Substitution-GT | Mutsgene-F and Mutsgene-R-TH-GT |
| Substitution-GA | Mutsgene-F and Mutsgene-R-TH-GA |
| Substitution-GC | Mutsgene-F and Mutsgene-R-TH-GC |
| Substitution-AC | Mutsgene-F and Mutsgene-R-TH-AC |
| Substitution-AG | Mutsgene-F and Mutsgene-R-TH-AG |
| Substitution-AT | Mutsgene-F and Mutsgene-R-TH-AT |
| Substitution-TC | Mutsgene-F and Mutsgene-R-TH-TC |
| Substitution-TG | Mutsgene-F and Mutsgene-R-TH-TG |
| Substitution-TA | Mutsgene-F and Mutsgene-R-TH-TA |
| gene30-Perfect DNA | gene30-F and gene30-R |
| gene30-Deletion | gene30-F and-R-QS |
| gene30-Insertion | gene30-F and-R-CR |
| gene30-Substitution | gene30-F and-R-TH |
| gene100-Perfect DNA | gene100-F and gene100-R |
| gene100-Deletion | gene100-F and-R-QS |
| gene100-Insertion | gene100-F and-R-CR |
| gene100-Substitution | gene100-F and-R-TH |

**Table S4. Extended application of the error-correction efficiency of eMBS across DNA substrate pools with 100 bp**

| **Type of DNA** | **70.4%** | | **87.0%** | |
| --- | --- | --- | --- | --- |
|  | **Before** | **After** | **Before** | **After** |
| **Perfect DNA** | **39970** | **40928** | **54773** | **56655** |
| **Deletion** | **7653** | **5946** | **3786** | **2034** |
| **Insertion** | **7682** | **4268** | **3856** | **1121** |
| **Substitution** | **1443** | **1169** | **820** | **469** |
| **Total error** | **16778** | **11383** | **8462** | **3624** |
| **Bases sequenced** | **56748** | **52311** | **65117** | **58397** |
| **Ratio of error-free** | **70.4%** | **78.2%** | **87.0%** | **93.8%** |

**Figure S1. Backbone root-mean-square deviation (RMSD) of the wild-type (WT) enzyme and the L157C/G233C-E38N mutant (Mut) complexes.** RMSD values are shown for the simulations of the wild-type (black) and mutant (red) enzymes bound to the three error-containing DNA substrates: (A) insertion-error complex, (B) deletion-error complex, (C) substitution-error complex.

**Figure S2. Band-shift assay of eMutS-L157C/G233C-E38N-biotin with homoduplexes versus single-base mismatch heteroduplexes in storage buffers containing 50-200 mM KCl.**

**Reference：**

1.	Zhang J, Wang Y, Chai B, et al. Efficient and Low-Cost Error Removal in DNA Synthesis by a High-Durability MutS. *ACS Synthetic Biology*. 2020;9(4):940-952. doi:10.1021/acssynbio.0c00079
